# Assessing the Utilization and Effectiveness of YouTube in Anatomy Education Among Medical Students: A Survey-Based Study

**DOI:** 10.7759/cureus.55644

**Published:** 2024-03-06

**Authors:** Swagatika Pradhan, Chinmaya Das, Dhiren K Panda, Biswa B Mohanty

**Affiliations:** 1 Genetics, Institute of Medical Sciences and Sum Hospital, Siksha "O" Anusandhan University, Bhubaneswar, IND; 2 Anatomy, Institute of Medical Sciences and Sum Hospital, Siksha "O" Anusandhan University, Bhubaneswar, IND

**Keywords:** youtube, web, online, medical students, human anatomy, cadaver

## Abstract

Introduction

Learning methodologies, particularly in medical education, are evolving with the integration of internet-based technologies into daily life. As a platform, YouTube has become a significant tool for studying human anatomy among medical students. This study aims to assess the utilization of YouTube in learning human anatomy, the types of audio-visual materials used, and the platform’s perceived effectiveness in understanding and memorizing anatomical information.

Methods

A cross-sectional questionnaire study was conducted among 200 medical students at a medical college over one year, of whom 195 completed the questionnaire and were included. The questionnaire addressed general YouTube usage, specific usage for medical studies and human anatomy, types of audio-visual materials used, and the perceived effectiveness of YouTube in understanding and memorizing anatomical information. Data were analyzed using IBM SPSS Statistics for Windows, Version 25.0 (IBM Corp., Armonk, NY) for Pearson’s chi-square test to determine statistical differences based on gender and year of study.

Results

The study cohort comprised 195 medical students (average age: 19.8±1.1 years), 62.6% females and 37.4% males. YouTube emerged as extensively utilized, with 94.5% of males and 96.7% of females reporting general usage and 91.8% of males and 89.3% of females utilizing it for medical studies. For human anatomy learning, 93.2% of males and 89.3% of females relied on YouTube. Among the audio-visual materials, PowerPoint presentations were most prevalent, favored by 46.5% of males and 41.8% of females. Regarding effectiveness, 82.1% of males and 83.7% of females affirmed YouTube's enhancement of anatomical understanding, with 89% of males and 85.3% of females acknowledging its aid in memorization. Additionally, 90.4% of males and 87.3% of females recommended YouTube as an anatomy learning tool. Despite observed gender-based preferences for specific content types, no statistically significant differences were discerned in YouTube's usage and perception across genders.

Conclusions

YouTube is a widely used and effective tool for the study of human anatomy among medical students, facilitating the understanding and memorization of anatomical information. While cadaver dissection remains an irreplaceable part of medical education, the addition of YouTube as a learning resource can enhance the educational experience. Future research should focus on the in-depth exploration of content satisfaction and the potential role of YouTube in the broader anatomy curriculum.

## Introduction

Anatomy, the science of the human body’s structure, is fundamental in medical education. It equips medical students with crucial knowledge for their future professional activities, including surgical operations, symptom interpretation, physical examinations, and the management of advanced imaging techniques for three-dimensional visualization [1,2]. Internet-based technology has ingrained itself into daily life and affects how medical students learn [3]. The integration of web-based technologies has expanded learning opportunities for human anatomy education beyond traditional classroom settings. The younger generation of students, who have grown up with web-based technologies like YouTube, have high expectations for the value of digital resources. The widespread availability of freely accessible information is a hallmark of the digital age [4,5]. YouTube is indeed becoming a popular platform for studying human anatomy due to its ease of use, user-friendly publishing environment, and the fact that it is a free service. Medical students have found YouTube to be a valuable educational tool for learning physical examination and medical topics, such as neurocranium bones, histology, and embryology [6-8]. YouTube videos have been found to be useful in supplementing medical education, providing additional information, and helping students prepare for exams [9]. However, it is important to ensure the quality and accuracy of the information in these videos, and expert educators should be involved in their creation [10]. While multimedia resources, including YouTube videos, can assist in teaching anatomy, they may not fully prepare students for the psychological impact of studying human cadavers. Therefore, educators should consider the limitations of video-based instructional materials and provide a comprehensive learning experience for students. Adding computer- and web-based activities to a gross anatomy curriculum has been found to have a positive effect on learning outcomes [11,12]. The use of immersive technologies, such as videos, can enhance visual engagement and improve understanding of 3D anatomical structures [13]. Visual aids, including medical imaging, plastic models, schematics, and anatomical imagery, can be effectively utilized in videos to maximize knowledge of anatomy [14]. The internet and user-generated content have become valuable resources for learning anatomy, reflecting a paradigm shift in recent years [15]. These resources provide accessible and interactive learning experiences, making anatomy education more enjoyable and engaging for students. The use of virtual reality (VR) technology and cooperative learning strategies has also shown promise in enhancing anatomy education outcomes and reducing motion sickness. Future research should further explore the optimal integration of VR and cooperative learning strategies in diverse course types. This study aimed to assess medical students’ utilization of YouTube in learning human anatomy, the types of audio-visual materials used, and the platform’s perceived effectiveness in aiding understanding and memorizing anatomical information.

## Materials and methods

Study design

This cross-sectional questionnaire study was conducted at the Institute of Medical Sciences and Sum Hospital, Bhubaneswar, over one year to evaluate the use of YouTube in learning human anatomy among first-year medical students. The institutional ethical committee approval was obtained before the study (Approval No. IMS/603/23/11/2021).

Participants and sampling

Out of 200 targeted first-year medical students, 195 were included after exclusions for incomplete responses, resulting in 122 female (62.6%) and 73 male (37.4%) participants. A convenience sampling method facilitated broad student body representation.

Questionnaire design

The structured questionnaire, distributed via Google Forms, comprised sections on demographic information (gender, year of study), general and specific YouTube usage for medical and anatomical studies, and types of audio-visual materials utilized. It also evaluated YouTube's perceived effectiveness in enhancing anatomical understanding and memorization using Likert scales and included open-ended questions for qualitative feedback.

Statistical analysis

Data were analyzed using IBM SPSS Statistics for Windows, Version 25.0 (IBM Corp., Armonk, NY). Pearson’s chi-square test assessed differences based on gender and year of study, with p<0.05 considered statistically significant. This approach aimed to identify patterns in YouTube usage and its educational impact among medical students.

## Results

The study encompassed feedback from 195 medical students, with a gender distribution of 122 females (62.6%) and 73 males (37.4%), as detailed in Table 1. The average age of participants was 19.8±1.1 years, indicating a young cohort primarily in their early academic stages.

**Table 1:** Demographic and Statistical Analysis of Participants.

Demographic Data	Details	P-Value
Mean age in years	19.8 ± 1.1	N/A
Gender distribution		
Male	73 (37.4%)	0.475
Female	122 (62.6%)	

General and educational use of YouTube

YouTube emerged as a pivotal resource among medical students for general and educational purposes. A significant majority, irrespective of gender, reported utilizing YouTube. General usage was high among all participants, with 94.5% of males and 96.7% of females engaging with the platform for various purposes. The use of YouTube as a study tool in medical education was also substantial, with 91.8% of males and 89.3% of females leveraging it for medical studies, showcasing its importance in their educational toolkit. Specifically, 93.2% of males and 89.3% of females utilized YouTube for anatomy learning, underscoring its value in understanding complex subjects.

Preferences for educational content on YouTube

The study explored preferences for different types of audio-visual materials on YouTube. PowerPoint presentations were favored by 46.5% of males and 41.8% of females, while cadaver dissection videos were utilized by 32.8% of males and 36.8% of females. These preferences indicate a diverse use of the platform for educational content, reflecting the varied learning styles among students.

Perceived effectiveness of YouTube in medical education

The effectiveness of YouTube in enhancing medical education was positively rated by the participants. A notable 82.1% of males and 83.7% of females reported an improvement in their understanding of anatomy through YouTube. Furthermore, 89.0% of males and 85.3% of females found it helpful for memorizing and recalling anatomical information. The recommendation of YouTube as a learning resource was also high, with 90.4% of males and 87.3% of females advocating for its use in studying anatomy.

Statistical analysis confirmed that there were no significant gender differences in the utilization and perceived effectiveness of YouTube for medical education (p>0.05 for all comparisons) (Table 2). This finding suggests that YouTube is a universally valuable tool for medical students across gender lines, enhancing their learning experience in a flexible and accessible manner.

**Table 2:** YouTube Usage in Anatomy Learning Based on Gender.

Questions	Male, n (%)	Female, n (%)	P-Value
Do you use YouTube in general?			
Yes	69 (94.5%)	118 (96.7%)	0.475
No	4 (5.5%)	4 (3.3%)	
Do you use YouTube as a source of information in medical study?			
Yes	67 (91.8%)	109 (89.3%)	0.628
No	6 (8.2%)	13 (10.7%)	
Do you use YouTube to learn anatomy?			
Yes	68 (93.2%)	109 (89.3%)	0.451
No	5 (6.8%)	13 (10.7%)	
What kind of audio-visual material on YouTube do you use to study human anatomy?			
Cadaver dissection videos	24 (32.8%)	45 (36.8%)	0.151
PowerPoint presentation	34 (46.5%)	51 (41.8%)	
Tutorial and illustrations	10 (13.6%)	15 (12.2%)	
Others	5 (6.8%)	11 (9.0%)	
Has using YouTube helped you to understand anatomy better?			
Yes	60 (82.1%)	102 (83.7%)	0.54
No	13 (17.9%)	20 (16.3%)	
Has using YouTube helped you to memorize and recall anatomical information?			
Yes	65 (89.0%)	104 (85.3%)	0.487
No	8 (10%)	18 (14.7%)	
Do you advise other students to use YouTube as a learning tool for anatomy?			
Yes	66 (90.4%)	107 (87.3%)	0.536
No	7 (9.6%)	15 (12.2%)	

## Discussion

The primary objective of our study was to examine the utilization of YouTube as a supplementary educational tool for anatomy learning among medical students and to assess whether there were any significant gender-based differences in its use. Our findings indicate that YouTube is a widely used resource among medical students, with a substantial majority of male and female students engaging with the platform for educational purposes. This high level of engagement underscores the importance of digital resources in contemporary medical education, particularly in studying complex subjects such as human anatomy [16-18].YouTube's diverse content, including PowerPoint presentations and cadaver dissection videos, was favored by students for its ability to complement traditional learning methods. The preference for visual aids and multimedia content reflects the evolving educational landscape, where digital and traditional resources merge to enhance the learning experience. This trend is supported by Baykan et al. (2007) [19], who highlighted the enduring value of cadaver dissection in anatomy education, suggesting that despite technological advancements, traditional methods remain integral to comprehensive anatomical understanding.

In our study, PowerPoint presentations emerged as a preferred audio-visual tool on YouTube for studying human anatomy, chosen by 46.5% of male and 41.8% of female students. Additionally, 32.8% of males and 36.8% of females utilized videos of cadaver dissections. These dissections remain a vital educational tool, appreciated for their effectiveness in elucidating the classification, relationships, and three-dimensional perspectives of human body parts, as well as for integrating theoretical and practical aspects of human anatomy, fostering touch-mediated perception, humanistic care, and practical skills application. The surge in human Anatomy videos on YouTube since its inception in 2005 can be attributed to the increased availability of the internet, advancements in video recording technology, and a shift towards self-directed learning in contemporary education [20-22].

The study also highlighted YouTube’s role in enhancing memorization, with 89% of male and 85.3% of female students acknowledging its benefits. Furthermore, a significant majority recommended YouTube as an anatomy learning tool, aligning with the notion that mastering anatomy requires a multifaceted approach involving comprehension, visualization, and memorization. This aligns with findings from a study indicating that videos on an anatomy YouTube channel can facilitate the creation of visual images [23,24].

While some research indicates gender-based differences in learning preferences and methodologies, others do not support this notion [25,26]. Our study observed gender differences in preferences for image-based videos and YouTube usage for learning specific anatomy areas. Our analysis revealed no significant gender differences in using YouTube for anatomy learning, suggesting that the platform is a universally accessible educational resource. This finding challenges previous research suggesting gender-based differences in learning preferences and methodologies [27,28]. Instead, our study contributes to the understanding that digital learning environments like YouTube may offer equitable learning opportunities across genders, supporting diverse learning styles and preferences.

Despite the promising insights, our study has limitations, notably the general nature of the questionnaire, which might have limited the depth of insights into specific YouTube content that enhances learning satisfaction and outcomes. Future research should explore the impact of dedicated anatomy YouTube channels on student learning experiences and outcomes. Investigating the potential of student-generated content to enhance engagement and retention, as well as examining anatomy educators' perspectives on integrating YouTube into the curriculum, could provide valuable insights into its efficacy as an educational tool.

## Conclusions

Our findings affirm YouTube's effectiveness as an educational tool for human anatomy, endorsed by over 90% of participants. The platform's resources, such as PowerPoint presentations and cadaver dissection videos, support anatomical learning across genders without significant differences in material selection or perceived effectiveness. Notably, our study found a slightly higher utilization rate of cadaver dissection videos among female participants (36.8%) compared to male participants (32.8%), although this difference did not reach statistical significance. This indicates broad uniformity in resource utilization across genders.

These results suggest YouTube's potential to democratize anatomy education, catering to diverse learning styles. Future research should further explore educational preferences and integrate digital resources like YouTube into the anatomy curriculum, enhancing learning outcomes for all medical students.
